# MicroRNA transcriptomic analysis of the sixth leaf of maize (*Zea mays* L.) revealed a regulatory mechanism of jointing stage heterosis

**DOI:** 10.1186/s12870-020-02751-3

**Published:** 2020-11-30

**Authors:** Gege Hou, Yahui Dong, Fangfang Zhu, Qiannan Zhao, Tianyi Li, Dandan Dou, Xingli Ma, Liancheng Wu, Lixia Ku, Yanhui Chen

**Affiliations:** College of Agronomy, State Key Laboratory of Wheat and Maize Crop Science, Henan Agricultural University, #15 Longzi Lake University District, Zhengdong New District, Zhengzhou, 450046 Henan People’s Republic of China

**Keywords:** Maize, miRNA, V6 stage, Sixth leaf, Photosynthesis, Jointing stage heterosis

## Abstract

**Background:**

Zhengdan 958 (Zheng 58 × Chang 7–2), a commercial hybrid that is produced in a large area in China, is the result of the successful use of the heterotic pattern of Reid × Tang-SPT. The jointing stage of maize is the key period from vegetative to reproductive growth, which determines development at later stages and heterosis to a certain degree. MicroRNAs (miRNAs) play vital roles in the regulation of plant development, but how they function in the sixth leaf at the six-leaf (V6) stage to influence jointing stage heterosis is still unclear.

**Result:**

Our objective was to study miRNAs in four hybrid combinations developed in accordance with the Reid × Tang-SPT pattern, Zhengdan 958, Anyu 5 (Ye 478 × Chang 7–2), Ye 478 × Huangzaosi, Zheng 58 × Huangzaosi, and their parental inbred lines to explore the mechanism related to heterosis. A total of 234 miRNAs were identified in the sixth leaf at the V6 stage, and 85 miRNAs were differentially expressed between the hybrid combinations and their parental inbred lines. Most of the differentially expressed miRNAs were non-additively expressed, which indicates that miRNAs may participate in heterosis at the jointing stage. miR164, miR1432 and miR528 families were repressed in the four hybrid combinations, and some miRNAs, such as miR156, miR399, and miR395 families, exhibited different expression trends in different hybrid combinations, which may result in varying effects on the heterosis regulatory mechanism.

**Conclusions:**

The potential targets of the identified miRNAs are related to photosynthesis, the response to plant hormones, and nutrient use. Different hybrid combinations employ different mature miRNAs of the same miRNA family and exhibit different expression trends that may result in enhanced or repressed gene expression to regulate heterosis. Taken together, our results reveal a miRNA-mediated network that plays a key role in jointing stage heterosis via posttranscriptional regulation.

**Supplementary Information:**

The online version contains supplementary material available at 10.1186/s12870-020-02751-3.

## Background

Heterosis is a phenomenon in which the vigour of a hybrid is superior to that of both of its homozygous parental lines; heterosis can be used to describe hybrid performance [1] and has played an important role in high-producing and high-quality agricultural products during the past century [2]. While its influence is indispensable in both plant and animal breeding, however, the molecular basis of heterosis is still unclear. Two major hypotheses concerning allelic heterozygosity were put forward to interpret the hereditary basis of heterosis: dominance [3, 4] and overdominance [5]. With the rapid development of genomic tools, nonallelic gene interactions have been intensively explored and are also considered a reason for heterosis; therefore, epistasis [6] and gene expression regulatory network hypotheses have been proposed. In recent years, different global expression trends [7, 8], specific protein functions, differential accumulation as well as posttranscriptional modification [9–11] have been identified as being related to heterosis of different organs and at different development stages at the molecular level.

Maize production represents the successful commercialized use of heterosis. Meanwhile, classification of heterotic groups improves the heterosis utilization efficiency; for example, Reid × Tang-SPT is a major heterotic pattern in China. Furthermore, a leading hybrid developed with the Reid × Tang-SPT pattern, Zhengdan 958, which has been widely planted across China during the last 20 years, has reached an estimated planting area of more than 43 million hectares to date. In addition, maize is also an appropriate model crop species for exploring the genetic mechanism of heterosis because this species includes many phenotypic, allelic [12], transcriptional [13, 14] and translational variations [15] and because its genomic information has been obtained [12, 16, 17]. The jointing stage of maize is a critical stage during which maize transitions from the vegetative stage to the reproductive stage and may affect development at later stages; thus, jointing stage heterosis can reflect grain yield heterosis to some extent. Plant leaves are sites where crucial biological functions occur, for instance, photosynthesis, respiration, transpiration and guttation [18]; in addition, compared with small leaves, larger leaves will give rise to increased photosynthesis yield, water- and nutrient-use efficiency, and biomass productivity [19]. Transcriptional and physiological metabolic processes were found to differ between super-hybrid rice and its parents, and the differentially expressed genes were significantly enriched in photosynthesis and carbon fixation [20, 21]. Comparing B73 × Mo17 with parental inbred lines, the gene with consistent high-parental or above high-parental pattern in at least two tissues was significantly enriched in photosynthesis [22]. All of these studies indicate that photosynthesis plays a key role in heterosis.

miRNAs are approximately 21-nt-long noncoding RNAs that negatively regulate gene expression at the post-transcriptional level [23]. Compared with the translational inhibitory component, miRNA-mediated cleavage of target messenger RNA (mRNA) seems to be the primary mechanism of posttranscriptional regulation [24]. Because of the advent of high-throughput sequencing technology, many miRNAs have been found in diverse plant species on a genome-wide scale, showing tissue-specific and/or development-dependent expression patterns [25–27]. In maize, multiple developmental processes are controlled by miRNA-mediated gene regulation; for instance, miR166 has been reported to regulate leaf polarity [28], overexpression of miR156 results in decreased expression levels of miR172 in the control of juvenile development [29], and miR172 regulates sex determination by targeting APETALA2 floral homeotic transcription factors (TFs) [30]. In addition, many studies have focused on the relationship between miRNAs and heterosis. Research has shown that the expression of some miRNAs in hybrid rice was compared with that in its parents and significant differences were identified [31]. In tomato, miRNA transcriptomes of seedlings of cultivated and wild species and their hybrids have been obtained [32]. A study in maize showed that in hybrid, miR167 is expressed at higher levels in 10-day-post-pollination kernels than in its parents, which suggested that miRNAs may participate in the regulation of heterosis [33]. Furthermore, most conserved miRNAs were more abundant in the parental inbred lines than in the hybrids, which implied that miRNAs in hybrids are generally repressed and may be responsible for heterosis of germinating maize embryos [34]. A study of multiple tissues or development stage from eight inbred parents and 12 hybrid genotypes revealed variation and inheritance pattern in sRNAs [35]. Together, all studies indicate that miRNAs play a key role in heterosis; nevertheless, how miRNAs and potential target genes function in jointing stage heterosis of maize is still unclear. In our study, we sought to elucidate the role of miRNAs and their interaction with their target genes in jointing stage heterosis of maize hybrids using next-generation sequencing technology by performing a miRNA transcriptomic analysis of the sixth leaf of four hybrid combinations and parental inbred lines at the V6 stage.

## Results

### Photosynthesis index of the sixth leaf at the V6 stage and grain yield of hybrid combinations and inbred lines

**Fig. 1:**
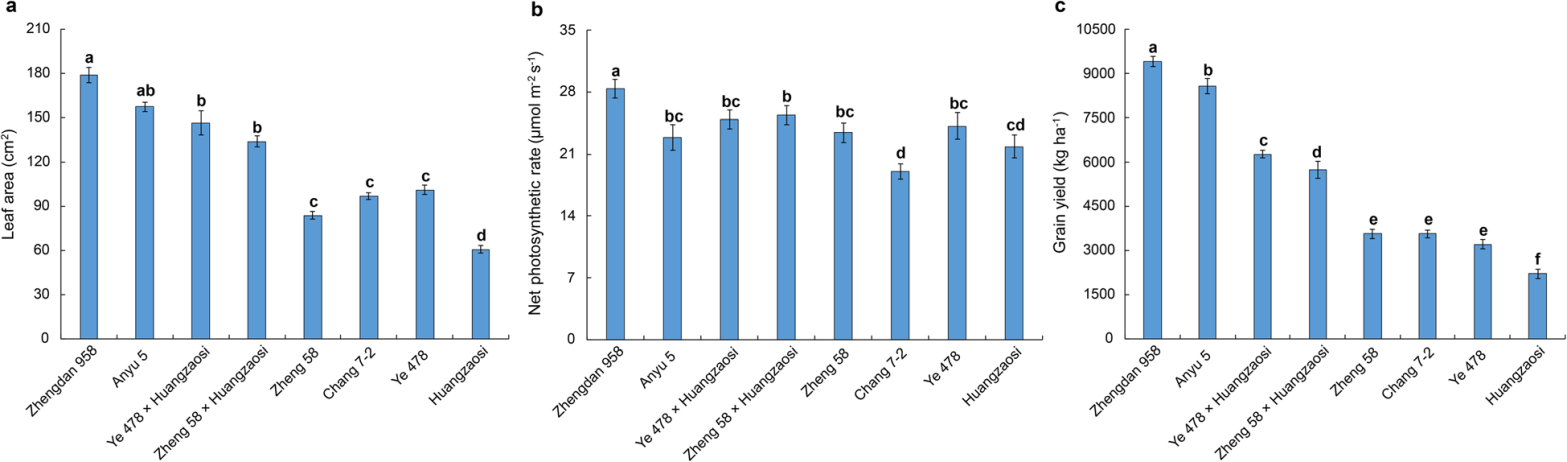
Characteristics of the leaf area, net photosynthesis rate of the sixth leaf at the V6 stage and grain yield of hybrid combinations and inbred lines. The lowercase letters indicate significant differences at *P* < 0.05 (least significant difference)

The four hybrid combinations were developed in accordance with the same heterotic pattern used in China. To assess the photosynthesis of these hybrid combinations and their parental lines, we measured the leaf area (Fig. 1a) and net photosynthesis rate (Fig. 1b) of the sixth leaf at the V6 stage when the sixth leaf had just fully expanded in the field. Furthermore, the grain field (Fig. 1c) was measured after harvest. The results showed that all hybrid combinations performed better than their parental inbred lines did, and each material presented distinctly different photosynthesis rates. We were interested in the potential miRNA regulatory network underlying jointing stage heterosis at the molecular level, so the miRNAs of the four hybrid combinations developed with the Reid × Tang-SPT pattern and their parental inbred lines were sequenced in the present study.

### miRNAs detected in the maize hybrid combinations and inbred lines

**Fig. 2:**
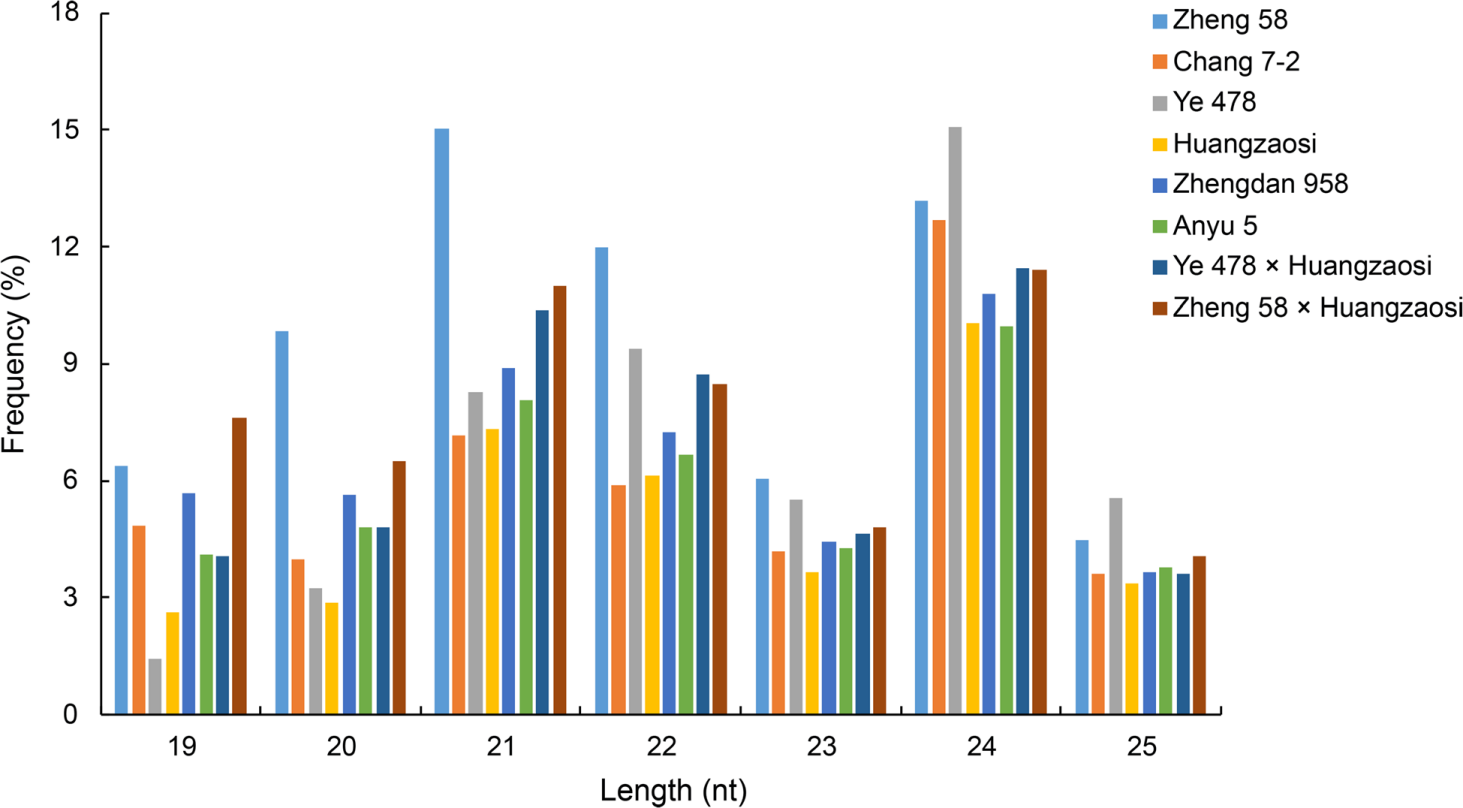
Length distribution of sRNAs in the sixth leaf at the V6 stage of the four hybrid combinations and inbred lines

sRNA pools of the sixth leaf at the V6 stage of the four hybrid combinations and inbred lines were sequenced via Illumina sequencing. After the low-quality reads were removed from the raw data, the clean data were further analysed. Analysis of the sRNA length distributions (Fig. 2) in the eight maize samples indicated peak sizes of 21 nt and 24 nt, and the sRNAs in the different categories were annotated (Table 1).

**Table 1:** The classification and annotation of small RNAs in inbred lines and hybrid combinations

Type	Ye 478		Zheng 58		Chang 7–2		Huangzaosi		Zhengdan 958		Anyu 5		Zheng 58 × Huangzaosi		Ye 478 × Huangzaosi	
	No.	(%)	No.	(%)	No.	(%)	No.	(%)	No.	(%)	No.	(%)	No.	(%)	No.	(%)
exon antisense	36,845	0.9	32,679	1.01	29,745	0.88	32,504	1.08	32,529	1	29,376	0.94	31,911	1.02	35,795	1.1
exon sense	351,662	8.63	310,750	9.6	267,915	7.91	252,898	8.41	281,694	8.69	363,730	11.62	318,596	10.22	246,757	7.6
intron antisense	65,219	1.6	58,022	1.79	53,706	1.59	50,573	1.68	54,489	1.68	49,937	1.59	53,732	1.72	58,784	1.81
intron sense	116,696	2.86	111,391	3.44	89,864	2.65	97,169	3.23	107,592	3.32	103,050	3.29	107,009	3.43	102,996	3.17
miRNA	1391	0.03	1720	0.05	1347	0.04	1476	0.05	1530	0.05	1529	0.05	1646	0.05	1684	0.05
rRNA	362,479	8.89	279,587	8.64	341,718	10.09	299,149	9.95	340,457	10.5	286,987	9.17	303,380	9.73	302,534	9.32
repeat	13,748	0.34	11,972	0.37	11,471	0.34	10,243	0.34	11,117	0.34	9977	0.32	10,564	0.34	11,587	0.36
snRNA	11,233	0.28	10,058	0.31	11,230	0.33	11,567	0.38	11,788	0.36	12,166	0.39	11,238	0.36	11,754	0.36
snoRNA	7598	0.19	7956	0.25	6524	0.19	7109	0.24	6330	0.2	6972	0.22	6376	0.2	6635	0.2
tRNA	122,738	3.01	68,283	2.11	103,502	3.06	90,753	3.02	86,469	2.67	79,262	2.53	72,217	2.32	82,897	2.55
unannotated sequences	2,987,131	73.27	2,343,364	72.42	2,470,517	72.93	2,154,506	71.63	2,307,998	71.19	2,188,282	69.88	2,201,162	70.6	2,384,698	73.46

**Fig. 3:**
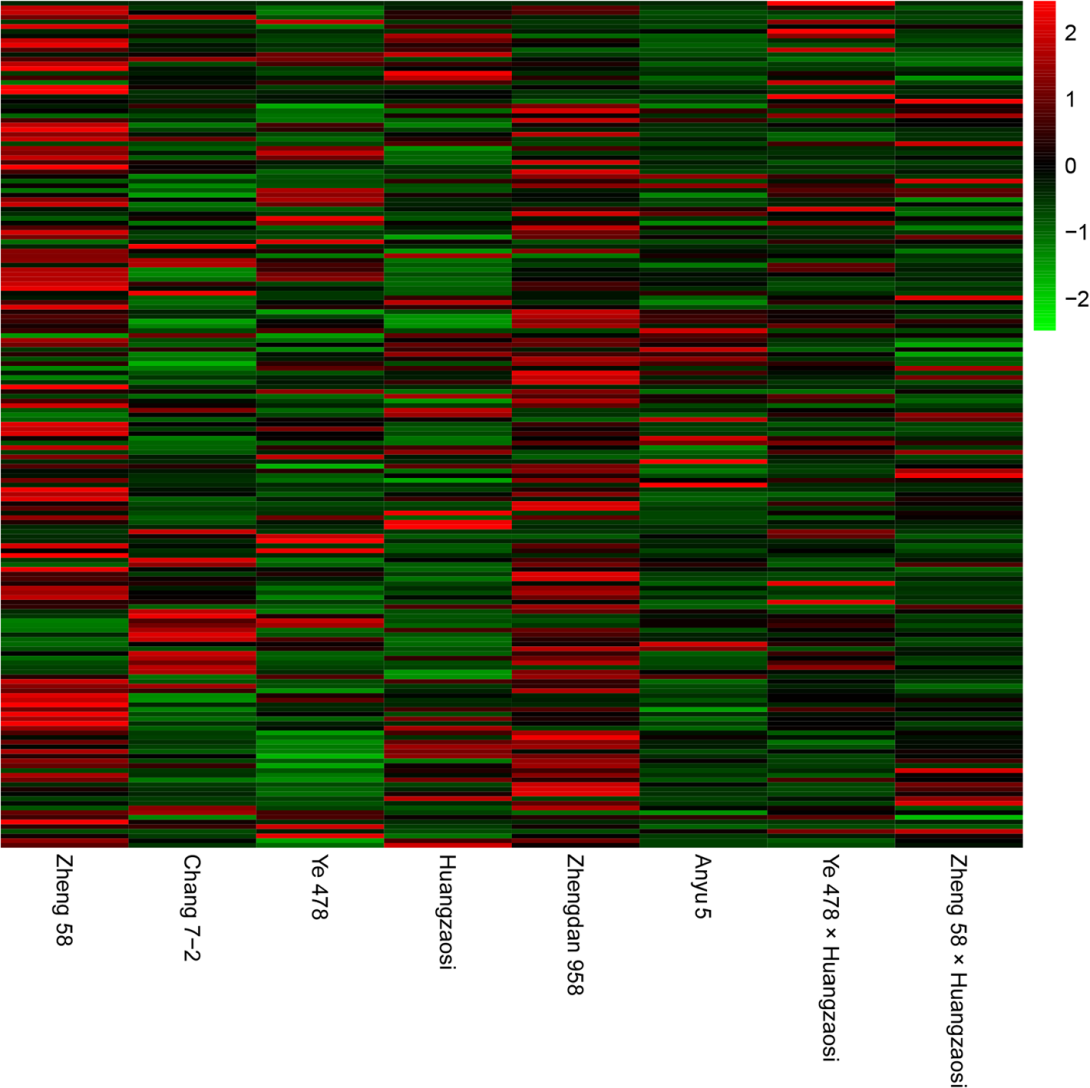
Expression of the total identified known miRNAs in the four hybrid combinations and the four inbred lines

With the exception of the sRNAs with unannotated sequences, sRNAs were annotated mainly as “rRNA” and “exon sense”. In total, there were 182 identified known miRNAs in miRBase (Fig. 3; Additional file 1: Table S1), and 52 novel miRNAs were identified (Additional file 2: Table S2). The number of reads for each miRNA was calculated and normalized to the number of transcripts per million (TPM). The expression pattern in the hybrid combinations was notably different from that in their parental lines.

### Expression pattern classification of the differentially expressed miRNAs in the sixth leaf at the V6 stage between the hybrid combinations and inbred lines

**Fig. 4:**
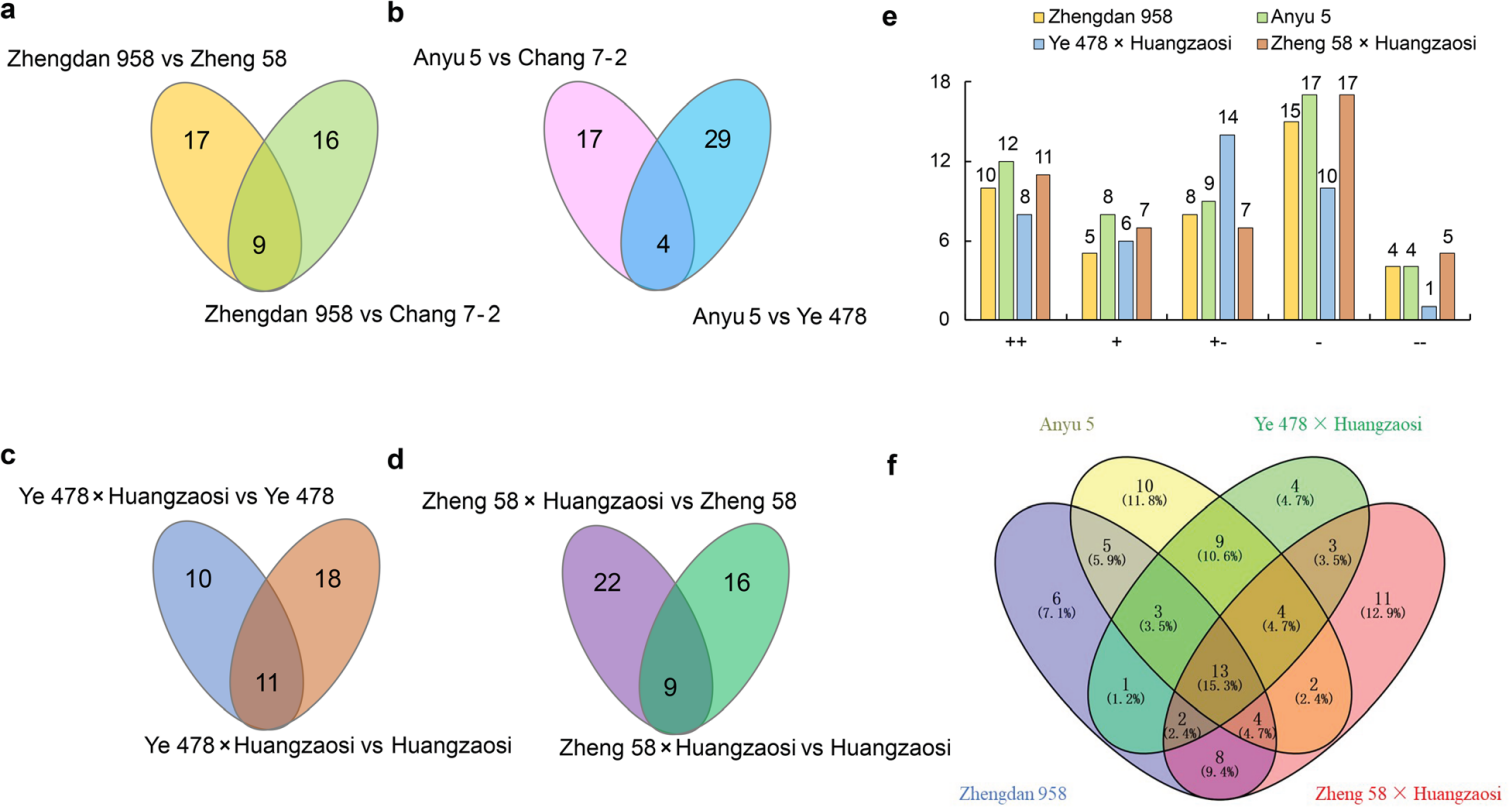
Characteristics of differentially expressed miRNAs between hybrid combinations and their parental lines and classification of the differentially expressed miRNAs. **a-d** Venn diagram of differentially expressed miRNAs between the four hybrid combinations and parental lines. **e** Expression pattern classification of the differentially expressed miRNAs in the four hybrid combinations. “++”, extremely high parental expression; “+”, high parental expression; “+−”, additive expression; “-”, low parental expression; “--”, extremely low parental expression. **f** Venn diagram of the expression patterns of scaled miRNAs in the four hybrid combinations

To classify the miRNAs into different categories, the miRNAs whose expression significantly differed between the hybrid combinations and parental lines were first identified according to the thresholds of *P* < 0.01 and log_2_(fold change) > 1 or < − 1. Twenty-six and 25 (9 in common) miRNAs were significantly differentially expressed between Zhengdan 958 and Zheng 58 as well as Zhengdan 958 and Chang 7–2, respectively (Fig. 4a; Additional file 3: Table S3–1). When Anyu 5 to Ye 478 and Chang 7–2 were compared, 33 and 21 (4 in common) miRNAs, respectively, were significantly differentially expressed (Fig. 4b; Additional file 4: Table S4–1), and 21 and 29 (11 in common) miRNAs were significantly differentially expressed between Ye 478 × Huangzaosi and Ye 478 and Huangzaosi, respectively (Fig. 4c; Additional file 5: TableS5-1). Similarly, 25 and 31 (9 in common) miRNAs were significantly differentially expressed between Zheng 58 × Huangzaosi and Huangzaosi and Zheng 58, respectively (Fig. 4d; Additional file 6: Table S6–1).

**Fig. 5:**
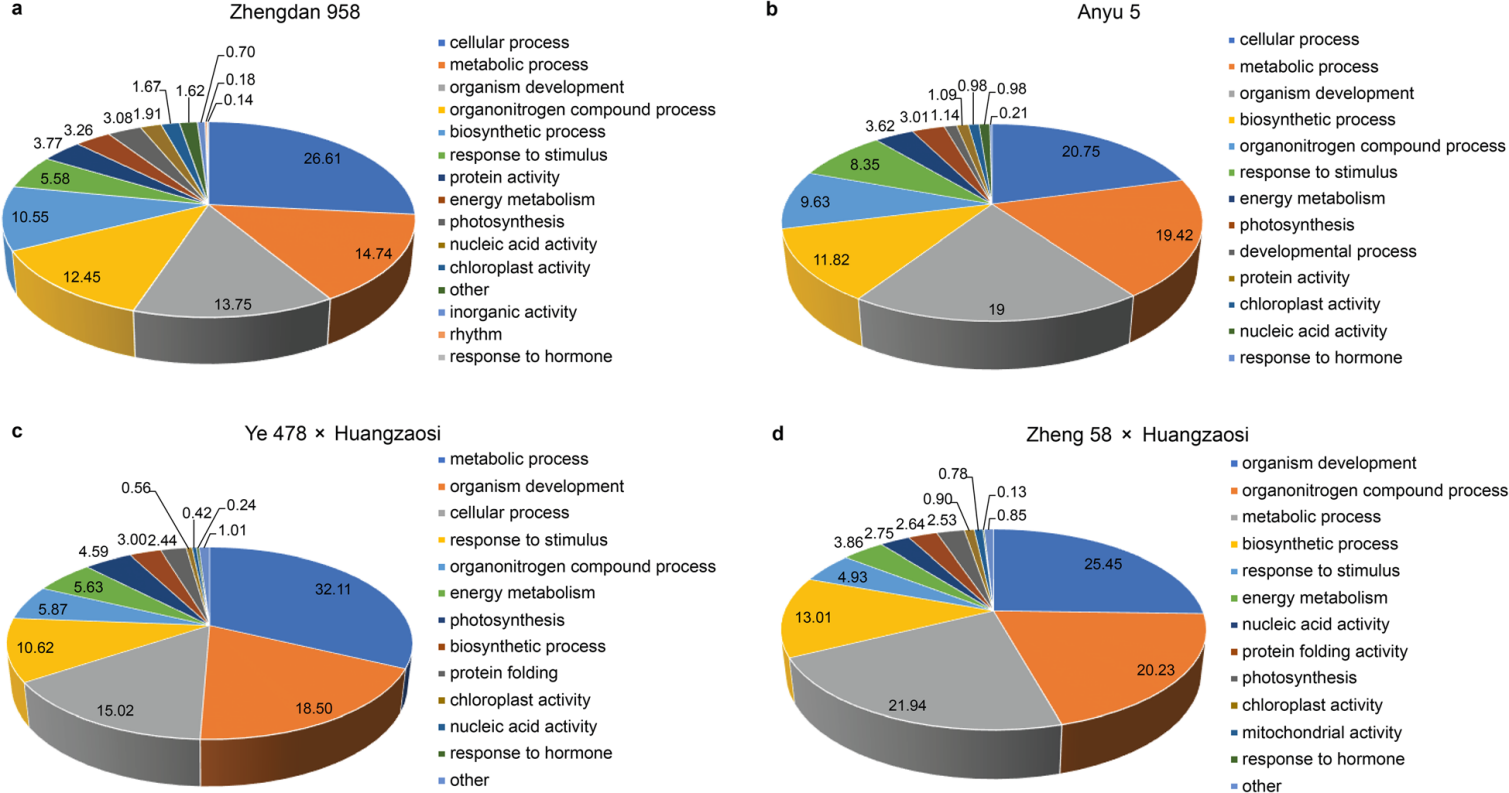
Major GO categories of biological processes for the target genes identified by the degradome of the scaled miRNAs. **a** Zhengdan 958; **b** Anyu 5; **c** Ye 478 × Huangzaosi; **d** Zheng 58 × Huangzaosi

A union of differentially expressed miRNAs in hybrid combination versus maternal inbred line and in hybrid combination versus paternal inbred line was further analysed. These 42, 50, 39 and 47 differentially expressed miRNAs in Zhengdan 958, Anyu 5, Ye 478 × Huangzaosi and Zheng 58 × Huangzaosi were classified into five distinct expression patterns according to the standard of classification mentioned in the methods; accordingly, these miRNAs are hereafter referred to as scaled miRNAs. The expression patterns could be explained by the additive, dominance, and overdominance hypotheses; most of the scaled miRNAs were non-additively (dominantly and over-dominantly) expressed. In total, 17, 23, 18, and 21 scaled miRNAs were induced (D/A > 0), while 25, 27, 21, and 26 were repressed (D/A < 0) in Zhengdan 958, Anyu 5, Ye 478 × Huangzaosi, and Zheng 58 × Huangzaosi, respectively, according to their D/A value. In general, miRNAs with low parental expression (−) constituted the most abundant miRNA group in Zhengdan 958, Anyu 5 and Zheng 58 × Huangzaosi; conversely, those with additive expression (+−) in Ye 478 × Huangzaosi constituted the most abundant miRNA group (Fig. 4e). According to the statistical data, 60, 54, 54, and 55% of the differentially expressed miRNAs were repressed in Zhengdan 958, Anyu 5, Ye 478 × Huangzaosi, and Zheng 58 × Huangzaosi, respectively. Within the biological process category, the major GO terms for the target genes of these scaled miRNAs in Zhengdan 958 (Fig. 5a), Anyu 5 (Fig. 5b), Ye 478 × Huangzaosi (Fig. 5c), and Zheng 58 × Huangzaosi (Fig. 5d) were enriched in photosynthesis, energy metabolism, chloroplast activity, and response to hormones, which indicates that these biological processes may contribute to heterosis.

### Characteristics of the scaled miRNAs in the sixth leaf at the V6 stage among the four hybrid combinations

There were 13 scaled miRNAs co-detected in the four hybrid combinations (Fig. 4f; Table 2), and some of these scaled miRNAs, including miR160b-3p, miR162-3p, miR162-5p, miR169c-3p and miR171g-5p, exhibited the same expression pattern in the four hybrid combinations.

**Table 2:** Expression pattern of the commonly expressed scaled miRNAs of the four hybrid combinations

miRNA ID	Zhengdan 958		Anyu 5		Zheng 58 × Huangzaosi		Ye 478 × Huangzaosi	
	D/A	classification	D/A	classification	D/A	classification	D/A	classification
zma-miR160b-3p	8.36	**++**	2.69	**++**	3.26	**++**	2.62	**++**
zma-miR166b-5p	5.38	**++**	−0.6	**–**	12.99	**++**	0.34	**+−**
zma-miR171g-5p	18.79	**++**	58.05	**++**	10.14	**++**	27.09	**++**
zma-miR169c-3p	1.67	**+**	1.9	**+**	0.9	**+**	1.44	**+**
zma-miR399d-5p	1.08	**+**	8.35	**++**	0.41	**+−**	2.87	**++**
zma-miR162-3p	−0.11	**+−**	−0.22	**+−**	− 0.2	**+−**	0.41	**+−**
zma-miR164e-5p	−0.39	**+−**	− 0.65	**–**	−0.4	**+−**	− 0.38	**+−**
zma-miR169f-3p	0.1	**+−**	0.74	**+**	0.33	**+−**	0.36	**+−**
zma-miR397b-3p	−0.47	**+−**	− 0.21	**+−**	−0.8	**–**	0.51	**+**
zma-miR167j-3p	−1.06	**–**	−0.36	**+−**	−0.34	**+−**	1.43	**+**
zma-miR162-5p	−0.82	**–**	− 0.74	**–**	− 0.82	**–**	−0.53	**–**
zma-miR395a-5p	−0.57	**–**	−2.14	**–**	2.33	**++**	− 0.19	**+−**
zma-miR1432-5p	−5.36	**–**	−0.92	**–**	−3.71	**–**	−1.14	**–**

Note: The D/A was calculated according to the formula (F1-MP)/(HP-MP) by expression abundance; “++”, extremely high parental expression (D/A greater than 2); “+”, high parental expression (D/A greater than 0.5 and below 2); “+−”, additive expression (D/A greater than − 0.5 and below 0.5); “-”, low parental expression (D/A greater than − 2 and below − 0.5); “--”, extremely low parental expression (D/A below − 2)

Six, 10, 4, and 11 scaled miRNAs were uniquely present in Zhengdan 958, Anyu 5, Ye 478 × Huangzaosi, and Zheng 58 × Huangzaosi (Fig. 4f). Some miRNAs exhibited interesting expression trends. Many miR156 members were differentially expressed in the hybrids, and different miR156 members were expressed in different hybrids. In Zhengdan 958, Anyu 5 and Zheng 58 × Huangzaosi, these members were mainly low parental or extremely low parental expressed, while most of the miR156 members c Ye 478 × Huangzaosi, with the exception of miR156j-3p, which had extremely low parental expression (Table 3). The miR395 members also displayed different expression biases in the different hybrid combinations. The scaled miR395b-3p was presented only in Zhengdan 958, Anyu 5 and Ye 478 × Huangzaosi; scaled miR395b-3p exhibited a “-” expression pattern in the first two hybrid combinations but showed a “+” expression pattern in the last combination (Table 4). miR395e-5p was uniquely present in Zhengdan 958 and exhibited a “+−” expression pattern, zma-miR395o-5p exhibited a “+−” expression trend in Zheng 58 × Huangzaosi and Ye 478 × Huangzaosi, and zma-miR395a-5p exhibited different expression patterns in all four hybrid combinations.

**Table 3:** Expression pattern of miR156 family members in four hybrid combinations

miRNA ID	Zhengdan 958		Anyu 5		Zheng 58 × Huangzaosi		Ye 478 × Huangzaosi	
	D/A	classification	D/A	classification	D/A	classification	D/A	classification
zma-miR156e-3p	**\**	\	\	\	−5.62	–	\	\
zma-miR156 h-3p	− 0.86	–	\	\	\	\	\	\
zma-miR156k-5p	−0.99	–	\	\	\	\	\	\
zma-miR156d-3p	−6.63	–	\	\	−1.56	–	\	\
zma-miR156l-3p	\	\	\	\	−0.75	–	−0.35	**+−**
zma-miR156j-3p	\	\	−3.19	–	−1.61	–	−5.11	–
zma-miR156a-5p	− 1.12	–	\	\	\	\	0.26	**+−**

Note: The D/A was calculated according to the formula (F1-MP)/(HP-MP) by expression abundance; “++”, extremely high parental expression (D/A greater than 2); “+”, high parental expression (D/A greater than 0.5 and below 2); “+−”, additive expression (D/A greater than −0.5 and below 0.5); “-”, low parental expression (D/A greater than − 2 and below − 0.5); “--”, extremely low parental expression

(D/A below − 2)

**Table 4:** Expression pattern of miR395 family members in four hybrid combinations

miRNA ID	Zhengdan 958		Anyu 5		Zheng 58 × Huangzaosi		Ye 478 × Huangzaosi	
	D/A	classification	D/A	classification	D/A	classification	D/A	classification
zma-miR395e-5p	−0.3	+−	\	\	\	\	\	\
zma-miR395a-5p	−0.57	–	− 2.14	–	2.33	++	− 0.19	+−
zma-miR395o-5p	\	\	\	\	0.18	+−	−0.06	+−
zma-miR395b-3p	−1.02	–	− 1.41	–	\	\	0.69	+

Note: The D/A was calculated according to the formula (F1-MP)/(HP-MP) by expression abundance; “++”, extremely high parental expression (D/A greater than 2); “+”, high parental expression (D/A greater than 0.5 and below 2); “+−”, additive expression (D/A greater than −0.5 and below 0.5); “-”, low parental expression (D/A greater than − 2 and below − 0.5); “--”, extremely low parental expression (D/A below − 2)

miR408a uniquely showed a “+” expression pattern in Zhengdan 958, while miR408b-5p uniquely exhibited a “--” expression pattern in Zhengdan 958 and Anyu 5. miRNA528a-5p was repressed in Anyu 5 and Ye 478 × Huangzaosi, while miR528a-3p exhibited a “-” expression pattern in Zhengdan 958 and Zheng 58 × Huangzaosi (Table 5). Members of the miR399 family were mainly induced in the four hybrid combinations (Table 6). Thus, based on these findings, the different expression bias of members from the same miRNA family may also be responsible for heterosis.

**Table 5:** Expression pattern of miR408 and miR528 family members in four hybrid combinations

miRNA ID	Zhengdan 958		Anyu 5		Zheng 58 × Huangzaosi		Ye 478 × Huangzaosi	
	D/A	classification	D/A	classification	D/A	classification	D/A	classification
zma-miR408b-5p	−8.08	–	−6.08	–	\	\	\	\
zma-miR408a	1.38	+	\	\	\	\	\	\
zma-miR528a-5p	\	\	−2.96	–	\	\	−1.09	–
zma-miR528a-3p	−1.72	–	\	\	−0.93	–	\	\

Note: The D/A was calculated according to the formula (F1-MP)/(HP-MP) by expression abundance; “++”, extremely high parental expression (D/A greater than 2); “+”, high parental expression (D/A greater than 0.5 and below 2); “+−”, additive expression (D/A greater than − 0.5 and below 0.5); “-”, low parental expression (D/A greater than − 2 and below − 0.5); “--”, extremely low parental expression (D/A below − 2)

**Table 6:** Expression pattern of miR399 family members in four hybrid combinations

miRNA ID	Zhengdan 958		Anyu 5		Zheng 58 × Huangzaosi		Ye 478 × Huangzaosi	
	D/A	classification	D/A	classification	D/A	classification	D/A	classification
zma-miR399e-3p	\	\	0.08	+−	\	\	\	\
zma-miR399c-5p	\	\	\	\	\	\	−0.06	+−
zma-miR399d-5p	1.08	+	8.35	++	0.41	+−	2.87	++
zma-miR399e-5p	13.25	++	\	\	4.52	++	\	\
zma-miR399d-3p	\	\	0.52	+	\	\	−0.33	+−
zma-miR399a-3p	\	\	1.87	+	\	\	−0.39	+−
zma-miR399b-3p	\	\	1.47	+	\	\	−0.78	–
zma-miR399f-3p	\	\	1.08	+	\	\	−0.39	+−
zma-miR399 g-3p	33.41	++	6.16	++	\	\	\	\
zma-miR399a-5p	11.18	++	5.39	++	\	\	2.06	++
zma-miR399j-5p	5.61	++	1.62	+	17.75	++	\	\
zma-miR399i-5p	\	\	−0.91	–	0.63	+	−0.66	–
zma-miR399b-5p	\	\	2.37	++	−1.08	–	−0.72	–

Note: The D/A was calculated according to the formula (F1-MP)/(HP-MP) by expression abundance; “++”, extremely high parental expression (D/A greater than 2); “+”, high parental expression (D/A greater than 0.5 and below 2); “+−”, additive expression (D/A greater than − 0.5 and below 0.5); “-”, low parental expression (D/A greater than −2 and below − 0.5); “--”, extremely low parental expression (D/A below − 2)

### Validation of selected miRNAs and their target genes identified via degradome analysis

**Fig. 6:**
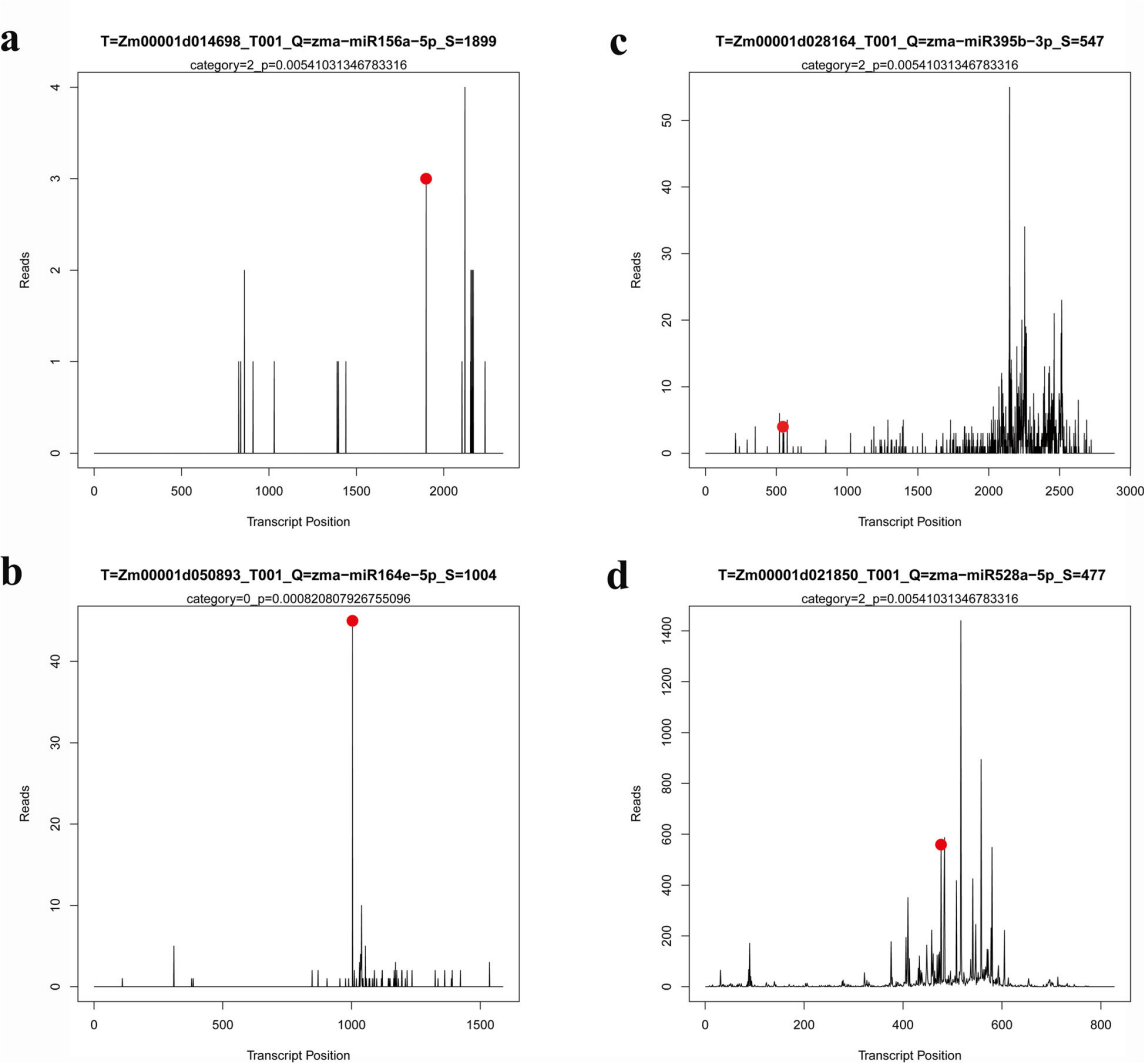
Examples of T-plots of miRNA targets confirmed by degradome sequencing. he T-plots show the distribution of the degradome reads along the full length of the target mRNA. The red point indicates the cleavage site of each transcript. **a** The cleavage features in SPL11 (Zm00001d014698_T001) mRNA by miR156a-5p. **b** Cleavage features in *NAC domain-containing protein 79* (Zm00001d050893_T001) mRNA by miR164e-5p. **c** Cleavage features in *sulfate transporter 2.2* (Zm00001d028164_T001) mRNA by miR395b-3p. **d** Cleavage features in a cupredoxin superfamily protein member (Zm00001d021850_T001) mRNA by miR528a-5p

**Fig. 7:**
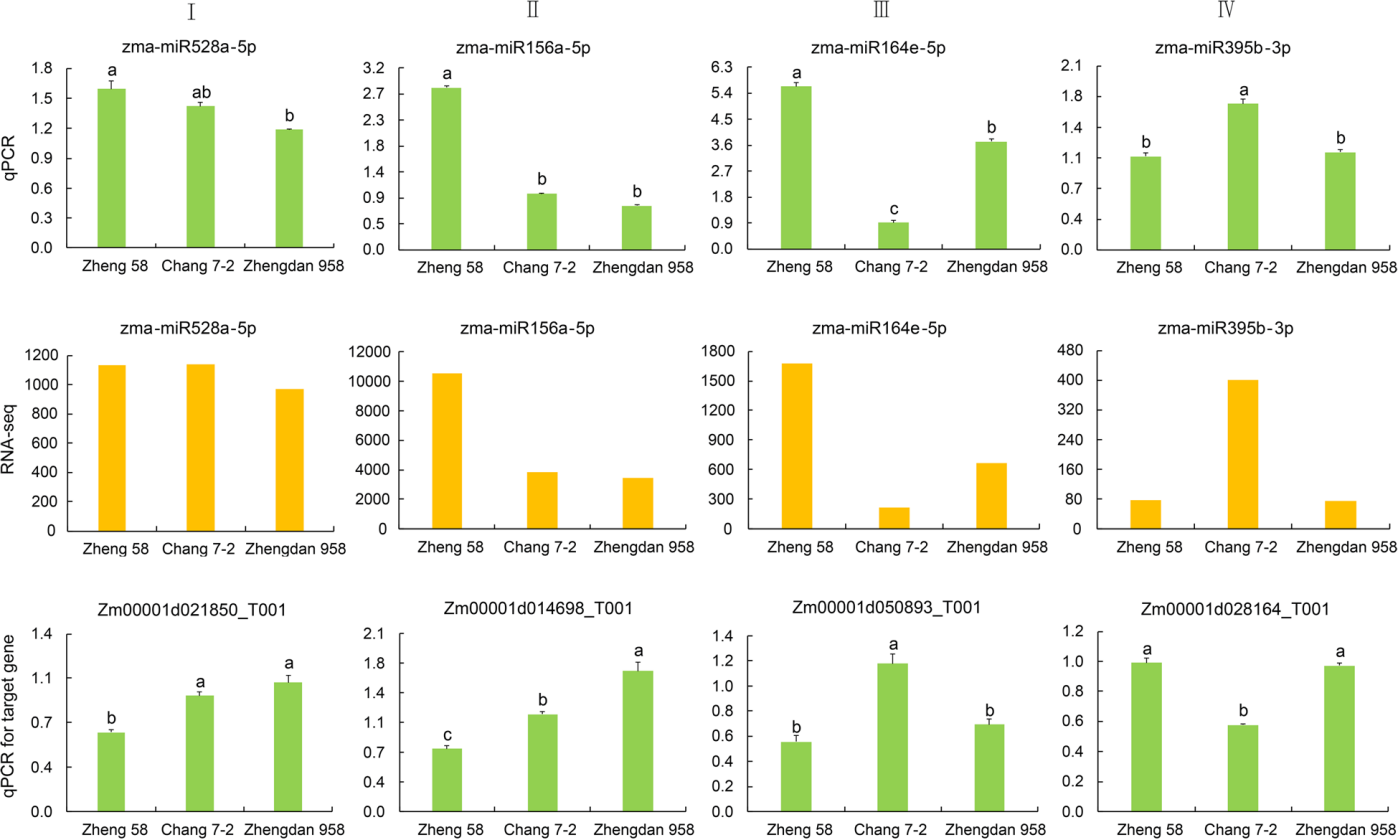
Verification of the expression patterns of selected miRNAs and their target genes in Zhengdan 958. The different lowercase letters above the columns indicate significant differences (*P* < 0.05)

Validating target genes is very important for understanding the biological function of miRNAs. In our research, we constructed a degradome library of the sixth leaf at the V6 stage. Target genes identified matched with scaled miRNAs in Zhengdan 958 (Additional file 3: Table S3–2), Anyu 5 (Additional file 4: Table S4–2), Ye 478 × Huangzaosi (Additional file 5: Table S5–2), and Zheng 58 × Huangzaosi (Additional file 6: Table S6–2). As may be expected, most transcripts targeted by conserved miRNAs were relative to conserved target genes. For instance, miR156a-5p targeted SPL11, miR164e-5p targeted NAC79, miR395b-3p targeted *sulfate transporter 2.2*, and miR528a-5p targeted cupredoxin superfamily proteins (Fig. 6). The expression profiles of these conserved miRNAs and target genes identified by the degradome were determined for the four hybrid combinations and their parental lines. We performed reverse transcription reactions for the total RNA, followed by quantitative real-time polymerase chain reaction (qRT-PCR). The internal control ZmActin was employed for qRT-PCR, and the 2^-ΔΔCt^ method was applied for expression-level calculation. The expression profiles of selected miRNAs were largely consistent with those from the sequencing data. Compared with the corresponding miRNAs, the target genes showed an opposite expression trend (Fig. 7; Additional file 7, 8, 9: Fig. S1, S2, S3).

## Discussion

### Relationships between heterosis, photosynthesis, and miRNAs

Heterosis, the superior performance of F1s relative to their parents, is prevalent for many traits and crosses of maize [36]. Maize displays dramatic heterosis for numerous traits, such as biomass, height, root growth, photosynthesis, starch metabolism, grain yield and biotic/abiotic stress resistance; hence, maize has served as a model plant for the study of heterosis. Zhengdan 958, a commercial hybrid that has a large planting area in China, represents the successful use of the Reid × Tang-SPT heterotic pattern. Recently, molecular and physiological proof has suggested that photosynthesis is associated with heterosis [37–39]. In *Arabidopsis*, whether in hybrid combinations or in their parents, if the rate of photosynthesis was constant per unit leaf, the hybrids presented an increase in total photosynthesis capability because of increased numbers of chloroplasts per cell, cell size and leaf area [19]. In maize, there are many studies regarding how miRNAs function in heterosis, such as studies pertaining to seed germination, internode expansion, and endosperm development [33, 40, 41]. We are interested in the potential mechanism by which miRNAs function in Zhengdan 958, Anyu 5 and the other two hybrid combinations of the Reid × Tang-SPT heterotic pattern to regulate jointing stage heterosis. Understanding this mechanism can help us better understand heterosis.

### Speculative miRNA-mRNA regulatory mechanism involved in jointing stage heterosis

In the present study, 182 known miRNAs were identified from the eight materials at the V6 stage, and the miRNAs differentially expressed in hybrids versus at least one parental line were scaled according to their D/A values. The expression of 25, 27, 21, and 26 miRNAs was repressed, while the expression of 17, 23, 18, and 21 miRNAs was induced, in Zhengdan 958, Anyu 5, Ye 478 × Huangzaosi, and Zheng 58 × Huangzaosi, respectively. Interactions between these scaled miRNAs and target genes may be related to heterosis. For instance, miR156, which is a class of star miRNAs reportedly involved in plant development and growth by binding specifically to the GTAC cis-element, target the SQUAMOSA promoter-binding-like (SPL) family of TFs [42, 43]. Given the increased knowledge regarding miR156, the miR156/SPL module has been suggested to be a multifaceted tool with which to enhance agronomic traits [44]. In rice, the os-miR156 and OsSPL14 modules control ideal plant architecture [45], and SPL TFs have also been found to modulate grain size, grain quality, panicle branching as well as plant height [46, 47]. In maize, miR156 was repressed in the hybrids, which may have induced SPL expression to increase internode expansion [41]. In our study, the repression of miR156a-5p may cause overexpression of *Squamosa promoter-binding-like protein 11* to accelerate the phase transition, which indicates that the hybrids have stronger growth potential than the parental inbred lines.

In maize inbred line B73, miR408b-5p has a single-nucleotide polymorphism (SNP) (A/G) at the 11th base position from the 5′end that differs from that of Mo17, which has been confirmed in another study, showing that parental expression between the B73 × Mo17 and Mo17 × B73 reciprocal crosses may cause functional variation [40]. The target genes of miR408b-5p identified by degradome, early light-induced proteins (ELIPs), which is a light-harvesting complex that binds chlorophyll and takes in solar energy in green plants in *Arabidopsis*. Suppression of the fast accumulation of ELIPs under intense light stress leads to leaf decolouration and causes extended photooxidative damage [48]. In our study, only the Mo17-allele miR408b-5p was downregulated in Zhengdan 958 and Anyu 5. Interestingly, miR408a, a miRNA that has been proven to result in improved biomass and seed yield by overexpression in *Arabidopsis* [49], was uniquely induced in Zhengdan 958. MIR408-overexpressing *Arabidopsis* plants presented remarkably improved leaf area, petiole length, plant height, flower size, and silique length as well as a significantly increased photosynthesis rate, which led to more biomass and seed yield. The genes whose expression was downregulated in *MIR408-OX* transgenic plants were remarkably enriched in pathways involving ribosomes, photosynthesis, carbon fixation, as well as pigment biosynthesis processes. In another study, miR408 overexpression resulted in increased fresh weight and increased root length at the seedling stage, and at the adult stage, all the leaves were enlarged [50]. In maize, miR408a upregulated in response to chilling stress to maintain leaf growth [51]. Based on our research, the high parental expression exhibited by miR408a only in Zhengdan 958 may also lead to increased photosynthesis and, thus, may have an effect on heterosis of maize hybrid combinations.

Sulfur is a mineral element essential to plant growth and is involved in photosynthesis, respiration, nitrogen and carbohydrate metabolism, thus regulating plant development [52]. Sulfate is the main form of inorganic sulfur available to plants; it is absorbed by plant roots and then translocated to diverse tissues for assimilation. In *Arabidopsis*, there are two forms of mature miR395 (miR395a,d,e and miR395b,c,f), which target two families of genes, the ATP sulfurylase and the *sulfate transporter 2;1*, both of which are involved in the sulfate metabolism pathway, and their transcripts are repressed strongly in miR395-overexpressing transgenic *Arabidopsis*, which have a relatively small stature and are slightly more chlorotic than the wild-type plants are. The mechanism involved in the maintenance of sulfate homeostasis by miR395 in plants has been elucidated [53]. Thus, we can infer that miR395b-3p is downregulated in hybrids to take advantage of sulfate more effectively.

Phosphate plays a key role in plant growth and development as an essential macronutrient, and it is a key structural component of nucleic acids, phospholipids, and the energy-carrying molecule ATP [54, 55]. miR399 family members participated in phosphate homeostasis as a phosphate deficiency-responsive factor in *Arabidopsis*. miR399 was shown to be induced under Pi deprivation to upregulate the splicing of the target mRNA PHO2 and suppress downstream PHT1 and PHO1 ubiquitination degradation by PHO2, promoting Pi absorption and transport [56–60]. In maize, miR399 expression is upregulated in response to Pi deficiency, and overexpression of miR399b leads to P accumulation in the shoots [61]. miR399 is also upregulated in the elongation zone, which may play possible roles in switching from cell division to cell elongation during leaf development [62]. In our study, miR399 was induced in hybrids, which may improve P-use efficiency to accelerate growth and development, which may ultimately be a cause of heterosis.

miR528s are a class of miRNAs restricted to monocots [63]. In rice, miR528 was proven to target at least four mRNA transcripts of genes that encode two plastocyanin-like proteins, an L-ascorbate oxidase and an EIN3-binding F-box protein [64]. Cu is an important element for protein synthesis [65]. There is an important class of Cu-containing proteins that function primarily as electron transfer proteins rather than as oxidases, which are commonly named blue Cu proteins or cupredoxins, such as plastocyanin-like proteins, which play an important role during photosynthesis [66]. In our study, miR528a-5p was repressed in the hybrid combinations, which may increase electron transfer to regulate photosynthesis.

The target of miR164 is the TF-encoding gene NAC1, which binds specifically to the cis-element IDE2. In many plant species, NAC TFs increase resistance to biotic and abiotic stresses by regulating the auxin signalling pathway [67–69]. Auxin can also promote NAC1 expression and functions downstream of TIR1 [70]; both auxin-induced miR164 and ubiquitination can decrease NAC1 transcription to reduce auxin signalling [71]. In our study, miR164e-5p was downregulated in the hybrid combinations; thus, NAC1 transcription may be upregulated, resulting in increased auxin signalling.

PS1-F (photosystem 1-F subunit) is a target of miR1432 identified by degradome in our study and has been demonstrated to regulate grain yield and tiller number in rice. Chlorophyll concentration and the electron transport rate were notably reduced in the homozygous Ds insertion OsPS1-F mutant line, leading to a reduction in plant height, tiller number, and grain yield, as well as pale yellow leaf coloration. If the mutant was complemented by proUBI::OsPS1-F, the phenotype due to the mutation was less pronounced [72]. In our study, miR1432-5p was repressed in the hybrids, inducing PS1-F, as a result, photosynthesis increased.

**Fig. 8:**
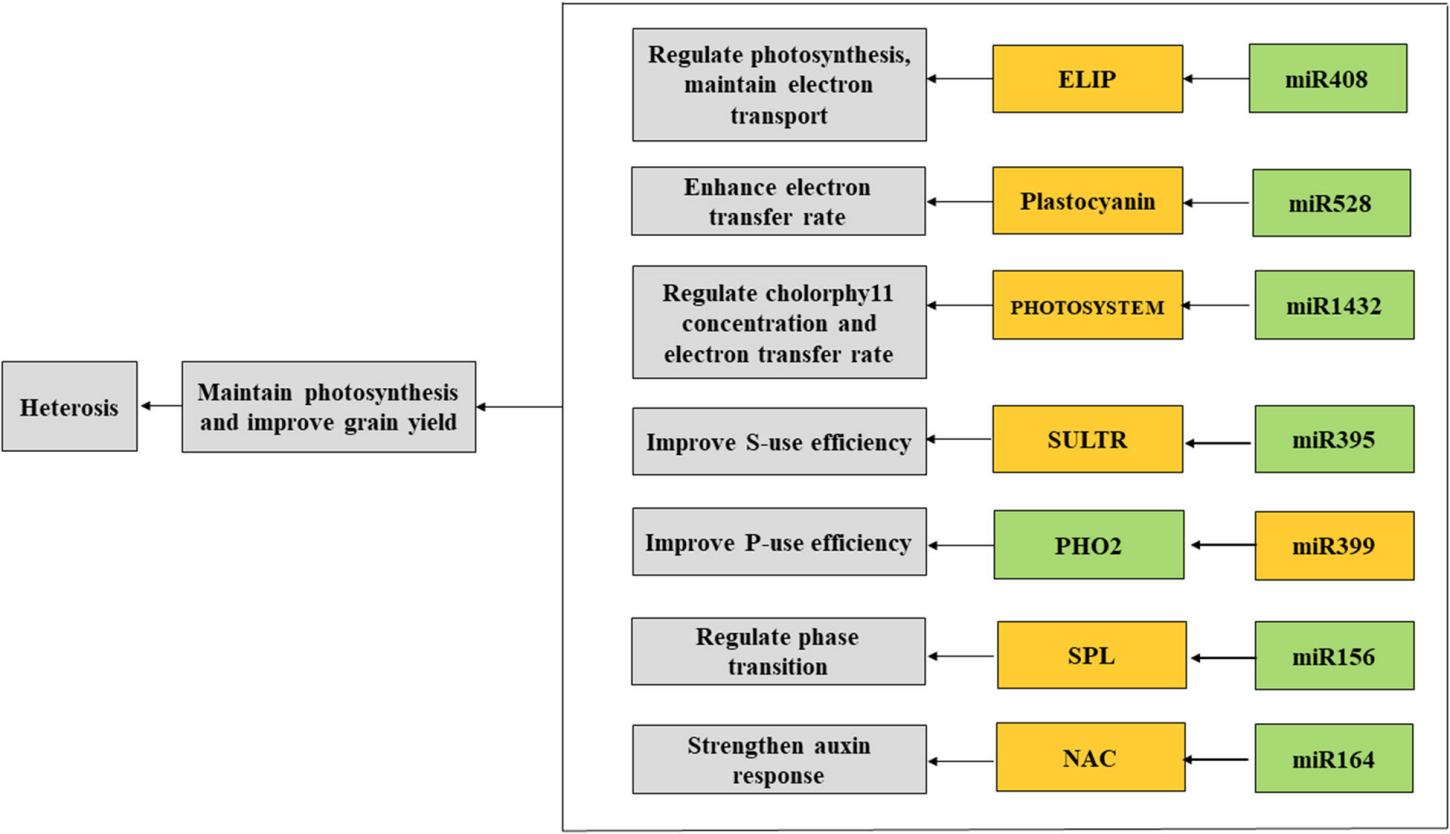
Model for miRNAs and their target genes associated with photosynthesis, hormone effects, and nutrient use. The black letters in the yellow block indicate upregulation, and the black letters in the green block indicate downregulation. The black arrows represent the direction of regulation. ELIP: early light-induced protein; SULTR, sulfate transporter; PHO2, PHOSPHATE 2; SPL, SQUAMOSA promoter-binding-like family transcription factors; NAC1, NAC domain-containing protein

The miRNA-mRNA modules may regulate jointing stage heterosis (Fig. 8). Among these miRNAs, the miR1432, miR164, and miR528 families were repressed in the four hybrid combinations; however, interestingly, the miR408, miR395, miR399, and miR156 families were expressed as different mature miRNA forms in the different hybrids and may exhibit different expression trends, which we believe may have different regulatory effects. Although many studies have worked on heterosis from different levels, such as gene expression, presence/absence variation, DNA methylation changes, protein changes as well as metabolite changes, our study focus on the miRNAs’ behaviour in hybrids and parents, which provide new knowledge about miRNA-mediated regulatory mechanism on heterosis. However, heterosis is a complicated, integrated scientific problem which still needs further studied.

## Conclusions

In this study, we found that 85 miRNAs were differentially expressed between the four hybrid combinations and parental lines at the V6 stage, and the union of the differentially expressed miRNAs was classified according to their D/A value. Of these scaled miRNAs, the expression of 25, 27, 21, and 26 miRNAs was repressed, while the expression of 17, 23, 18, and 21 miRNAs was found to be induced in Zhengdan 958, Anyu 5, Ye 478 × Huangzaosi, and Zheng 58 × Huangzaosi, respectively. Most of the scaled miRNAs were non-additively expressed. The scaled miRNAs from the same family expressed in different mature miRNAs exhibited different patterns in the different hybrid combinations, which may result in different posttranscriptional regulatory mechanism effects, followed by various heterosis phenotypes. We also validated the expression of some representative miRNAs by qRT-PCR. The potential miRNA target genes were confirmed via degradome sequencing. Our results indicate that a miRNA-mediated posttranscriptional regulation network, these scaled miRNAs (miR408, miR528, miR1432, miR395, miR399, miR156, miR164), may have roles in jointing stage heterosis via photosynthesis regulation. Our work provides useful information for further exploration of mechanisms involved in heterosis by miRNAs.

## Methods

### Plant materials

Zhengdan 958 (Zheng 58 × Chang 7–2), Anyu 5 (Ye 478 × Chang 7–2), Ye 478 × Huangzaosi, and Zheng 58 × Huangzaosi were developed in accordance with the popular heterotic pattern Reid × Tang-SPT in China, and their parental lines (Zheng 58 and Ye 478 are famous inbred lines from the domestic Reid group, and Chang 7–2 and Huangzaosi were famous inbred lines from the Tang-SPT group) were used as materials. We crossed the Zheng 58 and Ye 478 (maternal lines) with Chang 7–2, Huangzaosi (paternal lines) to gain four hybrid combinations, and the four hybrid combinations as well as the four inbred lines were then planted in the summer of 2018 on a farm at Henan Agricultural University (Zhengzhou, 113°42′E, 34°48′N) in northern China. In each replication, forty-five seeds of each material were planted in pots arranged in three lines in the field with 0.25-m line spacing and 0.60-m row spacing, such that the plant density was 67,500 ha^− 1^, and there were three replications.

### Photosynthesis index and grain yield measurements

When plants reached the V6 stage, the sixth leaf had just fully expanded, photosynthesis of the sixth leaf of 10 randomly selected seedlings was assessed in each replication for the eight materials by an LI-6400 photosynthesis system (LI-COR Biosciences, Lincoln, NE, USA) in the field between 8:00 a.m. and 11:00 a.m. Then, for the above-mentioned 10 selected seedlings, the length and greatest width of the sixth-fully-expanded leaf were used to measure leaf area according to the method of Montgomery [73]. In each replication, the middle row of each material was harvested to measure kernel weight per ear, and grain yield was calculated by the following formula: Grain yield (kg ha^− 1^) = kernel weight per ear (kg) × plant density (67,500 ha^− 1^). Three replications were applied.

### Maize RNA extraction, sRNA sequencing and miRNA identification

In each biological replication, equal amounts of tissue from the mid-part of the fully-expanded sixth leaf of 5 randomly selected seedlings from each material were harvested as soon as the plants reached the V6 stage and the sixth leaf had just fully expanded. They were stored at − 80 °C, and three replicates were obtained. Total RNA from each material was extracted using TRIzol reagent, and 24 sRNA libraries were constructed at Beijing Genomics Institute.

The raw data from Illumina sequencing were processed to filter and remove low-quality, repeats and low-complexity reads. To identify conserved miRNAs, unique sRNA sequences with lengths between 18 and 25 nt were mapped to miRNAs reported in miRBase 21.0 (http://www.mirbase.org/). After conserved miRNAs were annotated, the remaining parts of small RNA reads were used to identify novel miRNAs by the prediction software MIREAP (http://sourceforge.net/projects/mireap/). sRNAs that could map to the maize genome were considered potential miRNA candidates only if meeting the strict criteria reported in the literature [74].

### Identification of differentially expressed miRNAs and expression pattern classifications

The expression levels were normalized as TPM values. To evaluate statistical significance, a *t*-test was applied. Differentially expressed miRNAs between hybrid combinations and parental lines were identified with the following criteria: *P*-value was < 0.01 and log_2_(fold change) > 1 or < − 1. For each differentially expressed miRNA, the scaled difference was calculated as dominance/additivity (D/A value), based on TPM; the degree of dominance was calculated as hybrid - mid-parent; and the degree of additivity was calculated as high-parent - mid-parent. The D/A value was calculated by the formula: (F1-MP)/(HP-MP) [22]. A D/A greater than zero was considered induced, while a D/A less than zero was considered repressed. Scaled miRNA expression patterns were divided into five groups: (1) “+ +”, extremely high parental expression, with a D/A greater than 2; (2) “+”, high parental expression, with a D/A greater than 0.5 and less than 2; (3) “+−”, additive expression, with a D/A greater than − 0.5 and less than 0.5; (4) “-”, low parental expression, with a D/A greater than − 2 and less than − 0.5; and (5) “--”, extremely low parental expression, with a D/A less than − 2 [34].

### Degradome library construction and target identification

We equally mixed the 24 total RNAs used for miRNA sequencing to construct a single degradome library according to the method reported in the literature [75]. Single-end sequencing (50 bp) was performed on an Illumina HiSeq 2000 (Illumina, San Diego, CA USA). We used CleaveLand 3.0 to analyse the generated sequencing data. The identified target genes of the corresponding differentially expressed miRNAs were annotated with GO terms (http://www.geneontology.org/), which were considered significantly enriched when their *P*-value was < 0.05.

### Quantification of selected miRNAs and their target genes using qRT-PCR

We used a One Step PrimeScript miRNA cDNA Synthesis Kit (TaKaRa Co., Tokyo, Japan) to perform reverse transcription reactions according to the manufacturer’s instructions. qRT-PCR was performed with a SYBR PrimeScript miRNA RT-PCR Kit in conjunction with a fluorescence detection system (Roche LightCycler 480 II). The expression levels of the miRNAs were calculated using the 2^-ΔΔCT^ method (the primer sequences used are given in Additional file 10: Table S10). Differences in expression levels between hybrids and inbred lines were tested according to Fisher’s least significant difference test using Statistical Program for Social Science (SPSS) software; *P* < 0.05 was considered statistically significant.

## Supplementary Information

**Additional file 1: Table S1.** Known miRNAs identified in this study.

**Additional file 2: Table S2.** Novel miRNAs identified in this study.

**Additional file 3: Table S3.** Expression pattern of differentially expressed miRNAs in Zhengdan 958 and target function identified by degradome.

**Additional file 4: Table S4.** Expression pattern of differentially expressed miRNAs in Anyu 5 and target function identified by degradome.

**Additional file 5: Table S5.** Expression pattern of differentially expressed miRNAs in Ye 478 × Huangzaosi and target function identified by degradome.

**Additional file 6: Table S6.** Expression pattern of differentially expressed miRNAs in Zheng 58 × Huangzaosi and target function identified by degradome.

**Additional file 7: Fig. S1.** Verification of the expression patterns of selected miRNAs and their target genes in Anyu 5. The different lowercase letters above the columns indicate significant differences

**Additional file 8: Fig. S2.** Verification of the expression patterns of selected miRNAs and their target genes in Ye 478 × Huangzaosi. The different lowercase letters above the columns indicate significant differences (*P* < 0.05).

**Additional file 9: Fig. S3.** Verification of the expression patterns of selected miRNAs and their target genes in Zheng 58 × Huangzaosi. The different lowercase letters above the columns indicate significant differences (*P* < 0.05).

**Additional file 10: Table S10.** Oligonucleotide primers used for qRT-PCR assays in this study.

## Data Availability

The datasets generated and analysed during the current study are available in the NCBI Sequence Read Archive (SRA) database under Bioproject PRJNA649665 (https://www.ncbi.nlm.nih.gov/bioproject/PRJNA649665). The materials (Zheng 58, Ye 478, Chang 7–2, Huangzaosi, Zhengdan 958, Anyu 5, Ye 478 × Huangzaosi, Zheng 58 × Huangzaosi) used during the current study are available from the corresponding author on reasonable request.

## Abbreviations

miRNA: MicroRNA
sRNA: Small RNA
V6 stage: The six-leaf stage
GO: Gene ontology
qRT-PCR: Quantitative reverse transcription-polymerase chain reaction
SPL: Squamosa promoter binding protein-like
NAC: NAC domain-containing protein
ELIPs: Early light-induced proteins
PSII: Photosystem II complex
PS1-F: Photosystem-1 F subunit
PHO2: PHOSPHATE 2
PHO1: PHOSPHATE 1
PHT1: Phosphate transporter protein1
EIN3-binding F-box: ETHYLENE-INSENSITIVE3 transcription factors binding F-box
