# Supplementary figures and images for: MicroRNA transcriptomic analysis of the sixth leaf of maize (Zea mays L.) revealed a regulatory mechanism of jointing stage heterosis
Source: BMC Plant Biol. 2020 Nov 30;20:541. doi: 10.1186/s12870-020-02751-3 (PMC7708177; doi:10.1186/s12870-020-02751-3)

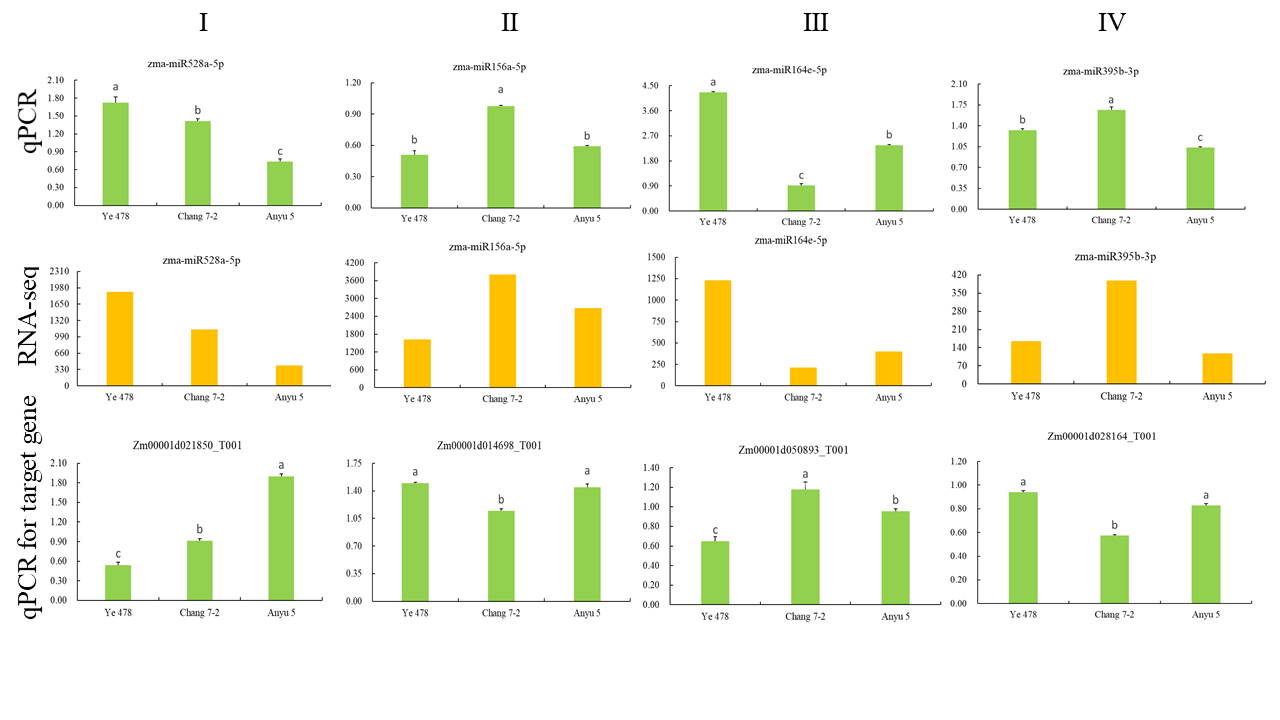

Supplement: Supplementary file 7 — Additional file 7: Fig. S1. Verification of the expression patterns of selected miRNAs and their target genes in Anyu 5. The different lowercase letters above the columns indicate significant differences [file 12870_2020_2751_MOESM7_ESM.tif]

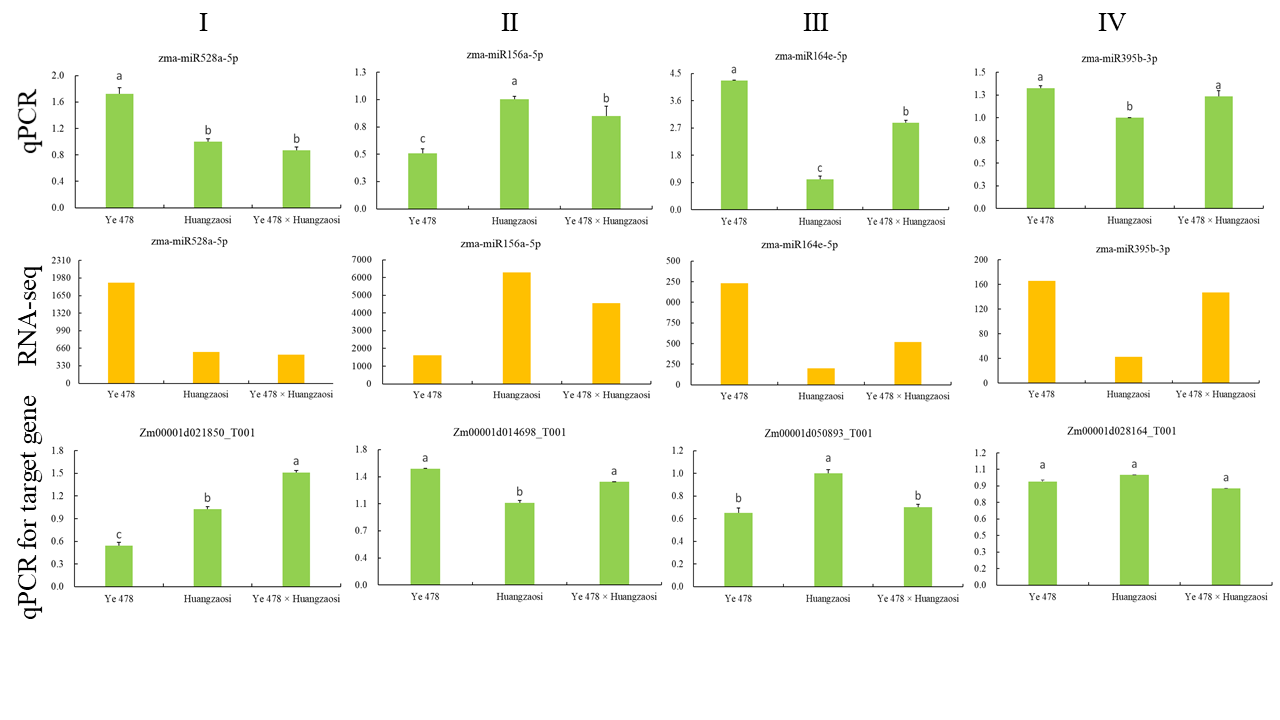

Supplement: Supplementary file 8 — Additional file 8: Fig. S2. Verification of the expression patterns of selected miRNAs and their target genes in Ye 478 × Huangzaosi. The different lowercase letters above the columns indicate significant differences (P < 0.05). [file 12870_2020_2751_MOESM8_ESM.tif]

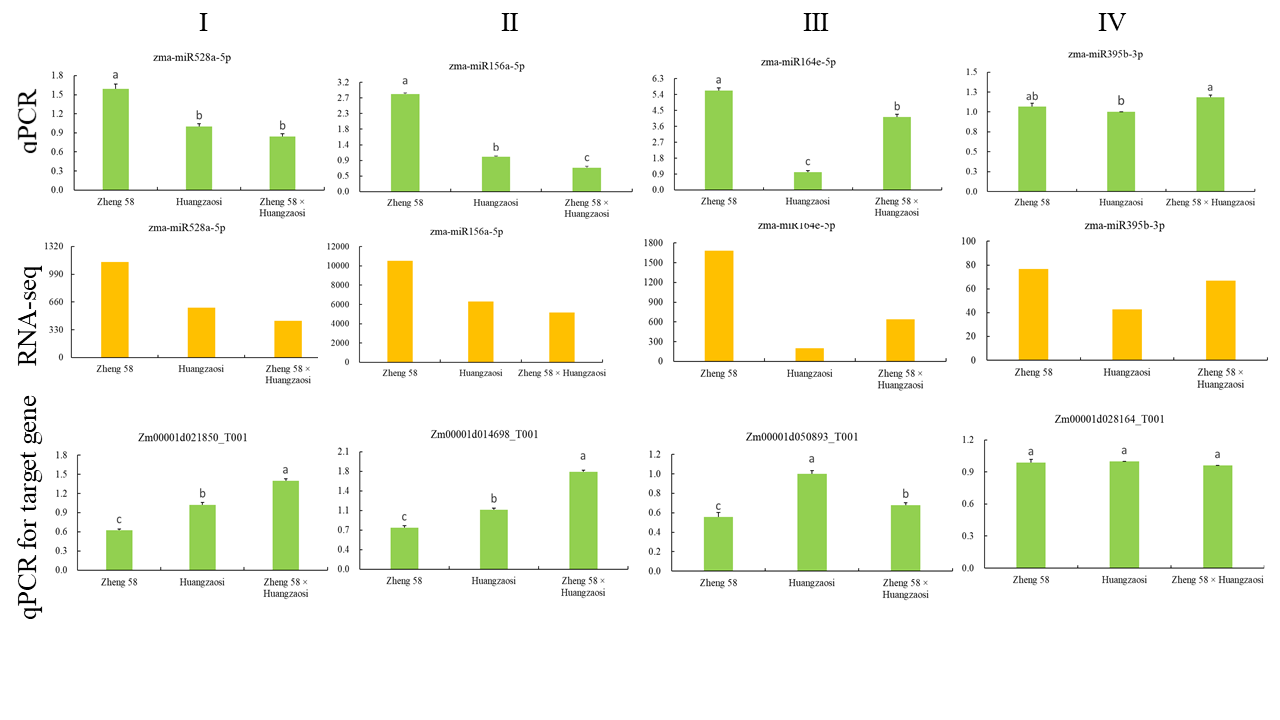

Supplement: Supplementary file 9 — Additional file 9: Fig. S3. Verification of the expression patterns of selected miRNAs and their target genes in Zheng 58 × Huangzaosi. The different lowercase letters above the columns indicate significant differences (P < 0.05). [file 12870_2020_2751_MOESM9_ESM.tif]
